# Mitochondrial accumulation of GRK2 as a protective mechanism against hypoxia-induced endothelial dysfunction

**DOI:** 10.1038/s41420-025-02628-0

**Published:** 2025-07-14

**Authors:** Cristina Gatto, Maria Rosaria Rusciano, Daniela Sorriento, Paola Di Pietro, Angela Carmelita Abate, Valeria Visco, Nicola Montone, Pasquale Mone, Daniele Di Napoli, Pierpaolo Chivasso, Vito Domenico Bruno, Vincenza Valerio, Paolo Poggio, Guido Iaccarino, Gaetano Santulli, Carmine Vecchione, Albino Carrizzo, Michele Ciccarelli

**Affiliations:** 1 University of Salerno “Scuola Medica Salernitana”, Department of Medicine, Surgery and Dentistry, 84081 Baronissi, Italy; 2 University of Salerno “Scuola Medica Salernitana”, Scuola di Specializzazione in Patologia Clinica e Biochimica Clinica, 84081 Baronissi, Italy; 3 Federico II University Hospital, Department of Advanced Biomedical Sciences, 80131 Naples, Italy; 4 University of Molise, Department of Medicine and Health Sciences “Vincenzo Tiberio”, 86100 Campobasso, Italy; 5 Einstein Institute for Aging Research, Einstein Institute for Neuroimmunology and Neuroinflammation, Albert Einstein College of Medicine, Department of Medicine, 10461 New York, USA NY; 6 Casa di Cura Montevergine, GVM Care and Research, 83013 Mercogliano, Italy; 7 University Hospital “San Giovanni Di Dio e Ruggi D’Aragona, Department of Emergency Cardiac Surgery, Cardio-Thoracic-Vascular, 84131 Salerno, Italy; 8 IRCCS Institute Galeazzi Sant’Ambrogio, Department of Minimal Clinical Cardio Surgery, 20157 Milan, Italy; 9 Cardiology Center Monzino IRCCS, 20138 Milan, Italy; 10 University of Milan, Department of Biomedical, Surgical and Dental Sciences, 20122 Milan, Italy; 11 Federico II University Hospital, Department of Clinical Medicine and Surgery, 80131 Naples, Italy; 12 International Translational Research and Medical Education (ITME) Consortium, 80100 Naples, Italy; 13 Einstein-Sinai Diabetes Research Center, Einstein Institute for Aging Research, Albert Einstein College of Medicine, Department of Molecular Pharmacology, 10461 New York, USA NY; 14 Mediterranean Neurological Institute Neuromed (IRCCS), 86077 Pozzilli, Italy

**Keywords:** Cardiovascular diseases, Translational research

## Abstract

Hypoxia, a condition characterized by a temporary lack of oxygen, causes mitochondrial damage, which in turn leads to endothelial dysfunction. G-protein-coupled receptor kinase 2 (GRK2) plays a key role in vascular homeostasis and remodeling, influencing endothelial function through various pathways. GRK2 moves within the cellular compartments and is linked to mitochondrial function and biogenesis, promoting ATP production and protecting against oxidative stress and cell death. The present study examined how mitochondrial GRK2 accumulation affects vascular reactivity and endothelial function in transient hypoxic conditions. Using a cloning strategy, we employed a small peptide (10aa) TAT-conjugated based on the pleckstrin homology domain of GRK2 to redirect GRK2 from the plasma membrane to the mitochondria. Mitochondrial accumulation of GRK2 increases vasodilatory responses in isolated swine artery segments, indicating potential therapeutic applications for cardiovascular disorders. Furthermore, in endothelial cells, GRK2 accumulation within mitochondria protects membrane potential, mitochondrial mass and prevents oxidative damage and cell death caused by transient hypoxia. Our findings show that GRK2 accumulation in mitochondria represents a potential therapeutic target to prevent transient hypoxia-induced damage.

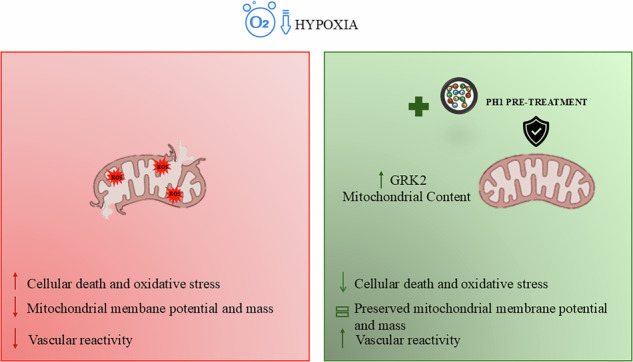

## Introduction

Hypoxia, a temporary oxygen deprivation condition, impairs oxidative phosphorylation and, thus, energy production [1]. It can be an acute or chronic phenomenon that affects specific tissues or the entire body. Oxygen monitoring is a critical mechanism that enables cells to detect and respond to changes in intracellular oxygen levels, which is necessary for physiological processes, including muscle activity [2]. Decreased oxygen levels trigger hypoxia responses, activating downstream pathways, including hypoxia-inducible factors, enhanced vascular permeability, and increased glycolytic enzymes [3, 4].

Hypoxia occurs in several pathophysiological situations (e.g., stroke for blood flow occlusion) [5] and activates downstream pathways associated with cardiovascular diseases (CVDs), including ischemia, heart failure, and atherosclerosis [6]. At the molecular level, mitochondria are the primary source of damage that increases oxidative stress and inflammation, leading to mitochondrial dysfunction [7].

Endothelial cells (ECs) are a single cell layer that lines blood vessels, regulates exchanges between the bloodstream and tissues, and modulates permeability, immune-mediated responses, and vascular tone [8]. Under physiological conditions, ECs produce nitric oxide, which promotes several processes that contribute to body homeostasis. Nevertheless, when the oxygen flow is interrupted, ECs experience severe stress. Although initially adaptive, prolonged hypoxia promotes endothelial dysfunction, oxidative stress, and increased blood vessel permeability, contributing to CVDs [9].

G-protein-coupled receptor kinases (GRKs) are a group of seven serine/threonine kinases divided into three subfamilies based on functional properties and sequence homology [10]. GRKs have a well-conserved catalytic domain (about 270 aa), an N-terminal domain (approximately 185 aa), and a variable-length carboxyl-terminal region (105–230 aa). The N-terminal domain plays a role in receptor identification, anchoring intracellular membranes, and includes an approximately 120 aa RH domain (regulator of G protein signaling homology domain). G protein-coupled receptor kinase 2 (GRK2) is a key regulator of G protein-coupled receptor (GPCR) signaling, and it is involved in cellular processes, including cell survival, proliferation, and inflammation [11–17]. The RH domain of GRK2 preferentially interacts with Gαq family members, preventing connection with phospholipase Cβ. The C-terminal portion of GRK2 has a pleckstrin homology domain (PH) that binds to free Gβγ subunits and the membrane phospholipid PIP2, essential for the protein agonist-dependent translocation to the plasma membrane [18, 19].

Although GRK2 was initially recognized for its role in the desensitization of GPCRs, mounting evidence suggests that GRK2 protects against stress-induced damage in CVDs [20]. As previously demonstrated, GRK2 regulates multiple mechanisms involved in vascular homeostasis and remodeling across various cell types [21], influencing endothelial function through the Akt/eNOS pathway [20].

βARK-ct is an engineered peptide that avoids GRK2 membrane translocation and activation by competing for membrane binding to Gβγ subunits of activated heterotrimeric G proteins (Gβγ) [21]. βARK-ct overexpression fosters GRK2 accumulation in the mitochondria [22, 23], improving function and biogenesis [24] and increasing ATP production for the ability to target and phosphorylate mitochondrial proteins [25]. According to Sato et al., GRK2 is in mitochondria and plays a role in energy metabolism [26]. Notably, it has been proposed that GRK2 accumulation in mitochondria following stress plays a protective role in mitigating oxidative stress and cell death [25, 27].

This study aims to investigate the protective role of mitochondrial GRK2 in hypoxia-induced endothelial dysfunction using a peptide based on the sequence of βARK-ct. We investigated the impact of mitochondrial GRK2 accumulation on oxidative stress and cell death in hypoxic endothelial cells. Furthermore, we analyzed the effect on vascular reactivity in isolated swine arteries, providing translational insights for potential therapeutic applications in CVDs.

## Results

### PH1 induces GRK2 accumulation in mitochondria

**Fig. 1:**
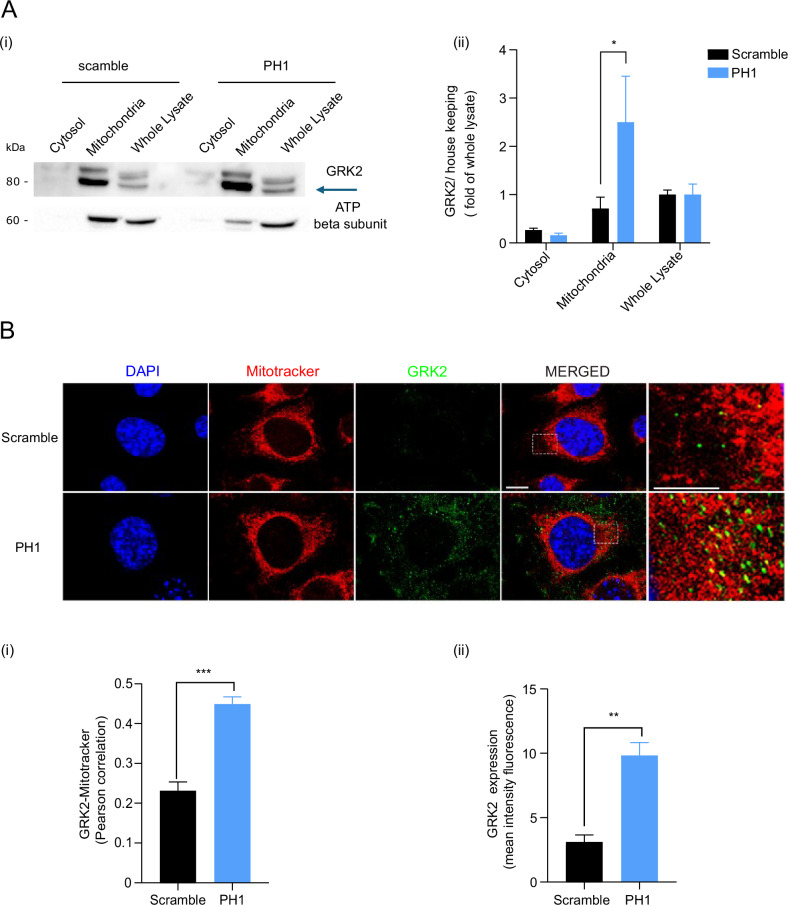
Mitochondrial localization of GRK2 in Endothelial Cells. In **A**, a representative immunoblot shows (i) the expression of G-protein-coupled receptor kinase 2 (GRK2) and ATP synthase β subunit in scramble and PH1-treated cells. The molecular markers are indicated on the left. Quantification (ii) shows band pixels between GRK2 and GAPDH for the cytosolic fraction and whole lysates, GRK2, and β subunit for the mitochondrial fraction. Data are the mean ± SEM of 3 independent experiments. *Two-way ANOVA*, *p < 0.05. In **B**, Immunofluorescence images showing co-localization of GRK2 (green) with mitochondria stained with MitoTracker (red) in ECs. Nuclei were counterstained with DAPI (blue). Merged images reveal areas of overlap between GRK2 and mitochondria (yellow), indicating mitochondrial localization of GRK2. (ii) Immunofluorescence analysis showing Pearson correlation of GRK2 (green) with mitochondria (red) in and (iii) GRK2 expression mean intensity fluorescence. Data are the mean of 3 independent experiments ± SEM. *p < 0,05, ***0,001 unpaired *test t-Student*.

The small peptide synthesized based on the carboxyl-terminal pleckstrin homology domain sequence of GRK2 induces the accumulation of GRK2 within the mitochondria. PH1 was obtained on a molecular cloning strategy to have the minimum sequence of βark-ct for enhancing GRK2 localization at mitochondria. The cytosol/membrane fractionation and immunofluorescence staining (Fig. 1A, B) show that PH1 causes an accumulation in the mitochondria, suggesting effective translocation.

### GRK2 enhances vascular reactivity in isolated swine vessels

**Fig. 2:**
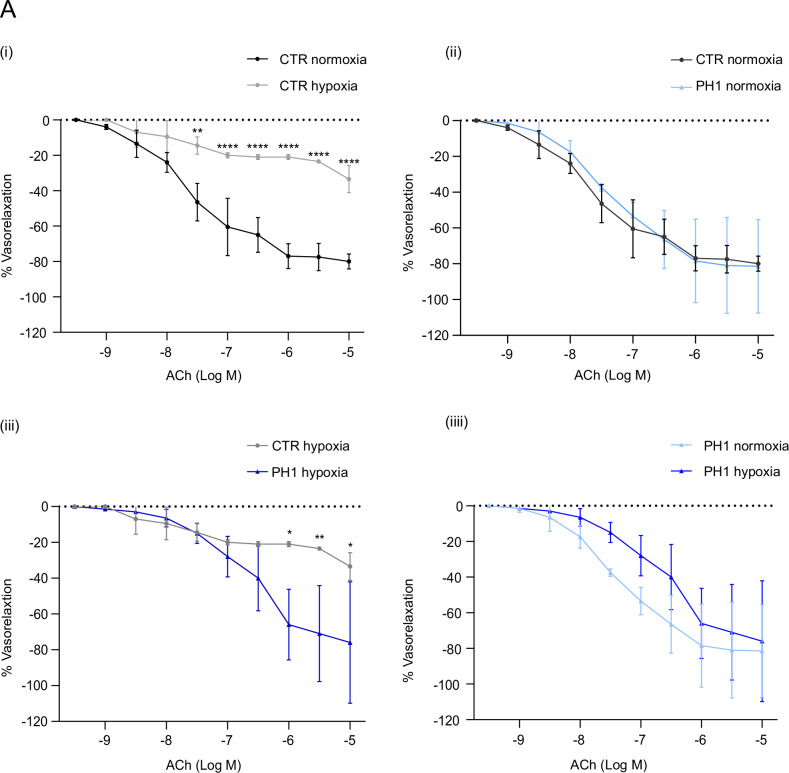
GRK2 improves vasodilation under hypoxia. In **A**, Vascular reactivity of the isolated femoral vessel in response to increasing doses of acetylcholine (Ach). Concentration–response curves for CTR and PH1-treated vessels are generated for normoxic and hypoxic conditions. Data are the mean of 3 independent experiments ± SEM. *Two-way ANOVA*, *p < 0.05, **p < 0,01.

During hypoxia, endothelial and vascular smooth muscle cells are susceptible to damage [28]. Reactivity studies show that hypoxia induces endothelial dysfunction, denoted by the attenuated vasodilatory responses to ACh in the CTR (Fig. 2A(i)), highlighting a progressive decline in function. Notably, treatment with PH1, thus the mitochondrial accumulation of GRK2 under basal conditions, does not impact endothelial function (Fig. 2A (ii)). This finding underlines the harmlessness of this treatment, an essential consideration for its translational potential.

**Fig. 3:**
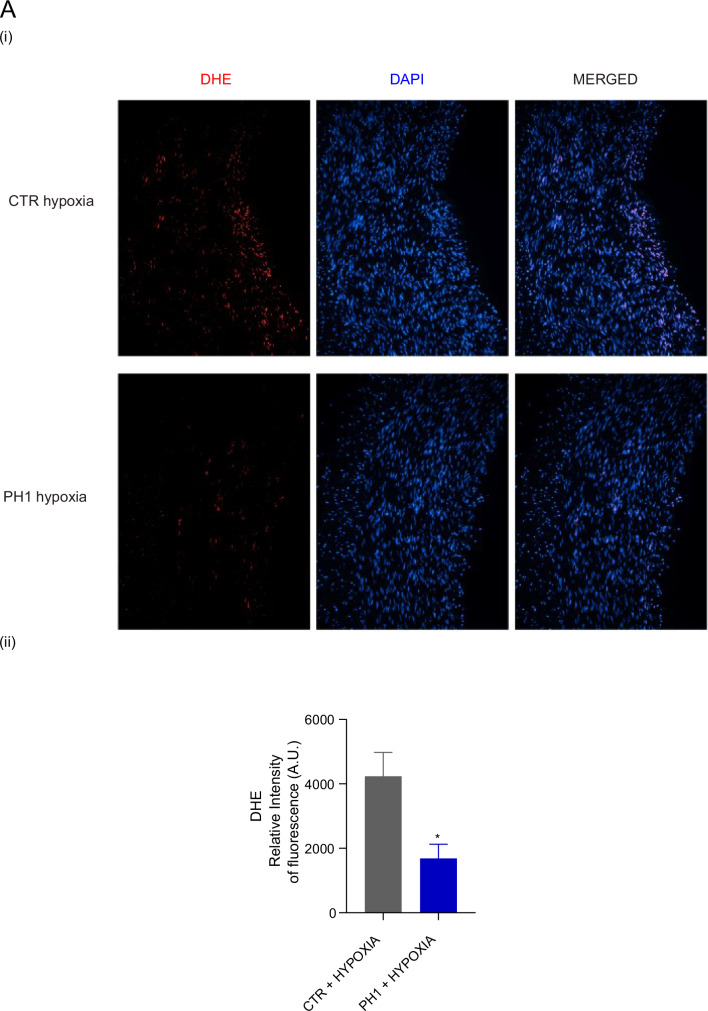
PH1 reduces vascular ROS in hypoxia. In **A** (i), representative images of ROS-positive cells stained with DHE, and the nucleus stained with DAPI. Quantification (ii) shows the red fluorescence (DHE) intensity in vessels under hypoxia conditions. Data are the mean of 3 independent experiments ± SEM. *p < 0,05 unpaired *test t-Student*.

After PH1 treatment, the compromised vasodilatory response to ACh is markedly recovered under hypoxia. A comparison of dose–response curves between the CTR and PH1 groups reveals an improvement in endothelial response to ACh, particularly at higher doses in hypoxic conditions. Thus, GRK2 accumulation in ECs enhances vasodilatory function, indicating an improved endothelial function (Fig. 2A(iii)). When compared under basal and hypoxic conditions, the PH1-treated groups show a similar trend for the curves, mirroring the protective impact of GRK2 translocation. The reduced ROS levels in pre-treated PH1 vessels demonstrate that GRK2 mitigates oxidative stress induced by hypoxia, contributing to improved vascular reactivity (Fig. 3A).

### Hypoxia induces increased HIF-1α levels in control cells under hypoxia

**Fig. 4:**
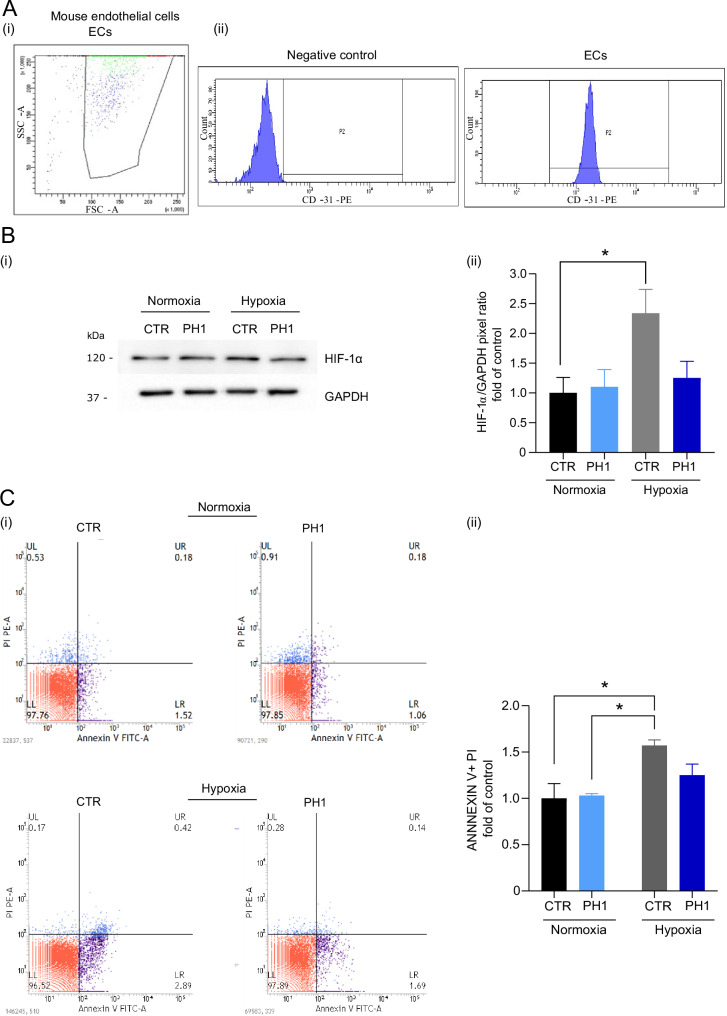
GRK2 limits hypoxia-induced cell death. In **A**, a representative image of CD31-positive cells demonstrating a high purity of ECs isolation. In **B**, the representative immunoblot shows (i) the expression of Hypoxia-inducible factor 1-alpha (HIF-1-α). Glyceraldehyde 3-phosphate dehydrogenase (GAPDH) is a loading control in CTR and PH1-treated cells in normoxic and hypoxic conditions. The molecular markers are indicated on the left. Quantification (ii) shows band pixels between HIF-1-α and GAPDH as fold of control (mean ± SEM of at least 3 independent experiments). *Two-way ANOVA*, *p < 0.05. In **C**, Annexin V/PI assay shows apoptotic levels in CTR and PH1-treated cells in normoxic and hypoxic conditions, with the representative plot (i). Histograms show (ii) the mean quantification ± SEM of apoptotic annexin V-positive cells (expressed as fold of control). Data are the mean ± SEM of at least 3 independent experiments. *Two-way ANOVA*, *p < 0.05.

The isolated ECs (Fig. 4A) were subjected to hypoxia as confirmed by the increase of HIF-1α, a crucial protein during hypoxia since it is primarily involved in oxygen deprivation response, triggering several adaptive pathways [29]. Hypoxia significantly increases HIF-1α levels in control cells (CTR), confirming hypoxia induction. In contrast, PH1-treated cells exhibited a lesser increase, indicating that GRK2 may regulate the HIF-1α stabilization (Fig. 4B).

### Mitochondrial accumulation of GRK2 attenuates hypoxia-induced cell death

Hypoxia from oxygen deprivation can trigger apoptosis. The degree of hypoxia determines whether cells undergo apoptosis or manage to adapt and endure [30]. CTR exposed to hypoxia exhibited an increase in apoptotic cells, whereas GRK2 accumulation in mitochondria resulted in a significant attenuation of hypoxia-induced cell death, as evidenced by reduced apoptotic cell population in PH1-treated cells (Fig. 4C), suggesting a possible protective effect due to GRK2 mitochondrial accumulation.

### Mitochondrial accumulation of GRK2 does not affect cell growth

**Fig. 5:**
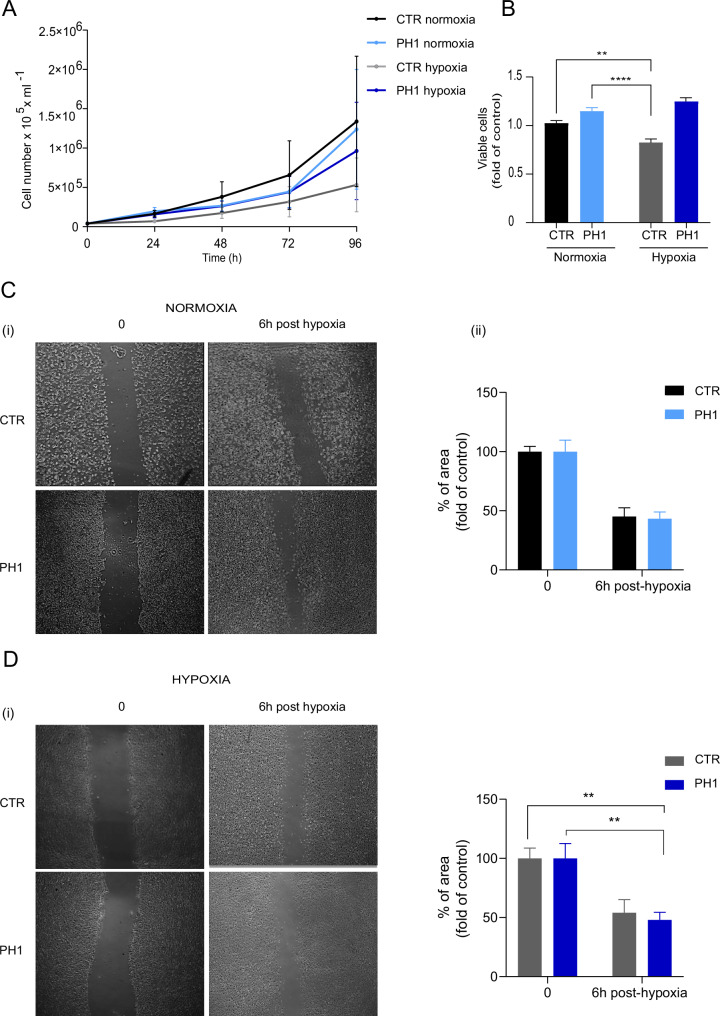
GRK2 preserves viability and promotes migration. In **A**, the growth curve of CTR and PH1-treated cells in normoxic and hypoxic conditions. Cells are detached and counted at 0, 24, 48, and 96 h. Data are the mean ± SEM of 3 independent experiments. In **B**, quantitative analysis of cell viability from the CCK-8 assay in normoxic and hypoxic conditions. The histograms represent the mean ± SEM of at least three independent experiments. In **C**, **D**, scratch test (i) demonstrates cell migration in CTR and PH1-treated cells in normoxic and hypoxic conditions, respectively. Histograms show (ii) the mean quantification of wound healing expressed as % of the area at 0 and 6 h post-hypoxia. Data are the mean ± SEM of at least 3 independent experiments. *Two-way ANOVA*, *p < 0.05.

Hypoxia is a well-established modulator of cell proliferation [31]; despite the different expressions of HIF-1α, the groups do not show significant differences in cell growth (Fig. 5A). The CTR exhibits a slowdown in growth under hypoxia conditions, as demonstrated by the 96-hour growth curve. Conversely, the PH1-treated cells, although subjected to hypoxia, show a similar growth trend to normoxic CTR cells. These data are confirmed in the cell viability assay, where a decrease in viability is observed in CTR cells subjected to hypoxia that is not evident in PH1-treated cells (Fig. 5B). Therefore, the accumulation of GRK2 in mitochondria could have a protective role in hypoxia-induced damage. However, PH1 treatment increases cell migration during the wound healing assay under hypoxic conditions. The scratch-induced wound size was similar between the groups basally, but PH1-treated cells showed a faster wound closure at 6 h post-hypoxia, indicating GRK2 plays a role within mitochondria (Fig. 5C, D).

### GRK2 reduces oxidative stress under hypoxic conditions

**Fig. 6:**
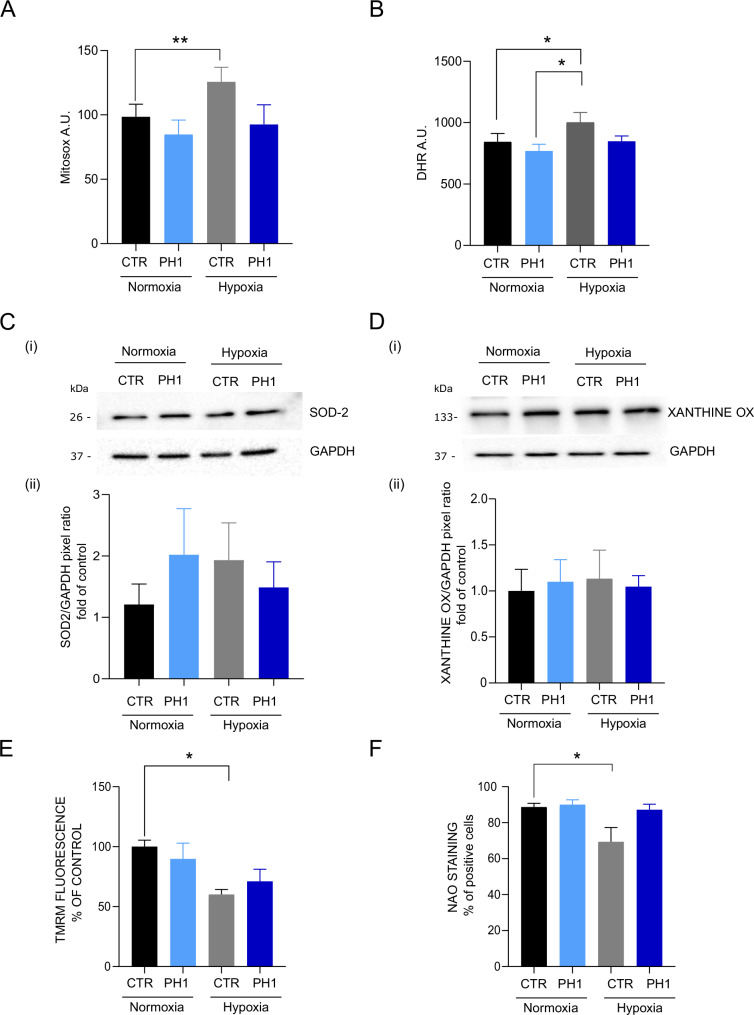
GRK2 reduces ROS and preserves mitochondria. In **A**, MitoSox shows mitochondrial superoxide production in CTR and PH1-treated cells in normoxic and hypoxic conditions. In **B**, the Dihydrorhodamine 123 (DHR) assay detects intracellular reactive oxygen species (ROS) in CTR and PH1-treated cells in normoxic and hypoxic conditions. Data are the mean of 3 independent experiments ± SEM. *Two-way ANOVA*, *p < 0.05, ** *p* < 0,01. In **C**, western blotting shows (i) the expression of mitochondrial superoxide dismutase (SOD-2), and in **D**, xanthine oxidase (xanthine ox). Glyceraldehyde 3-phosphate dehydrogenase (GAPDH) is a loading control in CTR and PH1-treated cells in normoxic and hypoxic conditions. The molecular marker is indicated on the left. Quantification (ii) shows band pixels between SOD-2, xanthine oxidase, and GAPDH (mean ± SEM of at least 3 independent experiments). In **E**, histograms show TMRM fluorescence monitoring the mitochondrial membrane potential in control and treated cells expressed as % of control. In **F**, the % of Nonyl acridine orange positive cells is a marker of mitochondrial mass. Data are the mean of at least 3 independent experiments ± SEM. *Two-way ANOVA*, *p < 0.05.

Hypoxia and mitochondrial dysfunction induced by hypoxia cause an overproduction of ROS, causing damage to DNA, lipids, and proteins, potentially leading to cell death [32]. Hypoxia induces oxidative stress as demonstrated by the increased intracellular and mitochondrial ROS levels in CTR exposed to hypoxia (Fig. 6A, B). Even during hypoxia, PH1 treatment protects cells from oxidative damage, resulting in lower ROS levels, confirming the data ex vivo. ROS production generally accompanies increased antioxidant defenses to restore the damage, primarily in mitochondria. SOD-2, although not statistically significant, increases in the CTR subjected to hypoxia but also in PH1-treated cells in normoxia, suggesting that mitochondrial GRK2 induces an increase in antioxidant defenses basally, thus allowing for oxidative mitigation (Fig. 6C). Xanthine oxidase, a ROS source in vascular tissue, is activated during hypoxia and contributes to oxidative stress and endothelial dysfunction [32]. However, no differences were observed among the groups (Fig. 6D).

### GRK2 preserves mitochondrial mass and membrane potential

Hypoxia is accompanied by mitochondrial mass reduction [33]. The NAO staining indicates that hypoxia induces a decrease in mitochondrial mass in the CTR, not evident in PH1-treated cells (6F), highlighting a protective role of mitochondrial GRK2 against acute damage, as demonstrated by Franco et al. [34]. A decrease in membrane potential mainly accompanies ROS increase as a marker of mitochondrial damage. TMRM results show that hypoxia induces a significant reduction in membrane potential in CTR and, thus, in energy production that is not evident in the PH1-treated group (6D). The membrane potential reduction correlates well with oxidative stress under hypoxia. The differences in membrane potential could be due to the maintenance of the activity of mitochondrial complexes by GRK2, since there are no differences in OXPHOS proteins (Fig. S1A) and mitochondrial biogenesis marker PGC-1α expression (Fig. S1B).

## Discussion

GRK2 is a serine/threonine kinase primarily identified on the plasma membrane where it binds and phosphorylates GPCRs, triggering the receptor’s desensitization and controlling GPCR activities. In addition, the GRK2 interactome has been extended to various signaling molecules and numerous cellular functions [35]. Recent evidence suggests GRK2 regulates several cellular activities, including proliferation and migration in ECs [36].

ECs, a thin layer inside the vascular blood vessels, are recognized as an active metabolic and endocrine organ [37] that can alter blood flow in response to changes in oxygen concentration, such as during hypoxia. Hypoxia, caused by reduced oxygen availability, activates several responses at the endothelial level [3], and alteration in these mechanisms results in endothelial dysfunction, impaired vascular reactivity, which underlies many CVDs.

Our study demonstrates an endothelial protective function for GRK2 translocation in mitochondria under hypoxia. The enhanced vascular reactivity in isolated swine vessels suggests that GRK2 may have therapeutic implications, improving vascular function in diseases characterized by endothelial dysfunction. Physiological levels of ROS are essential for endothelial homeostasis and smooth muscle cell contraction; nevertheless, excessive ROS induces endothelial dysfunction as ROS activates various protein kinases [38]. The ability of GRK2 to reduce oxidative stress, confirmed by in vitro studies, contributes to an intact vascular system and homeostasis. Besides, GRK2 removal increases ROS production in the endothelium by releasing cytokines and causing arterial wall inflammation [39]. Therefore, reducing ROS levels is an essential strategy to restore vascular function. Ex vivo studies suggest the potential role of GRK2 modulation in preserving endothelium-dependent vasodilation induced by Ach during hypoxia, offering a novel treatment approach for endothelial dysfunction that is not based on drug therapies.

Specifically, the mitochondrial accumulation of GRK2 is identified as a critical mechanism for counteracting hypoxia-induced oxidative stress and cell death. GRK2 is localized in mitochondria under basal conditions, but stressor events modulate the amount [40]. Depending on the physio-pathological context, GRK2 has either protective or detrimental effects. While in chronic conditions, such as heart failure, the increase of GRK2 affects cardiac function [41], in the acute setting as hypoxia, the kinase displays a different role. Particularly within mitochondria, GRK2 has a protective and pro-regenerative role after an acute or stressful event [22, 34]. Recent studies demonstrated that its mitochondrial localization is facilitated by interactions with heat shock protein 90 and modification as phosphorylation on specific residues (Ser670), preserving mitochondria from acute stress, such as ionizing radiation [38].

In this study, the mitochondrial accumulation of GRK2 induced by the peptide PH1 before an acute event as hypoxia, preserves ECs from damage due to attenuation of oxidative stress [22]. Consequently, this attenuation enhances cell survival, providing significant insights into the potential of PH1 as a therapeutic strategy to prevent hypoxic damage. The ability of PH1 to regulate GRK2 localization represents a promising therapeutic strategy for conditions where hypoxia and oxidative stress play critical roles, such as ischemia, neurodegeneration, or CVDs.

Mitochondria are key organelles that regulate metabolic flows in the cell [42]. Since ATP and ROS are generated in mitochondria, these organelles must be strictly controlled to react quickly to changes in external conditions or stresses [43]. ROS levels generally accompany hypoxia due to reduced phosphorylation efficiency and, thus, decreased oxidative phosphorylation. PH1 Treatment preserves mitochondrial membrane potential, as confirmed by the TMRM assay, indicating improved mitochondrial function. This effect likely reflects enhanced coupling between oxidative phosphorylation and ATP synthesis, reducing ROS generation. PH1 does not affect mitochondrial biogenesis or OXPHOS complexes under hypoxia, suggesting that its benefits are mediated by mitochondrial functional optimization rather than structural modifications.

Hypoxia induces mitochondrial dysfunction, resulting in mitophagy and oxidative stress [44]. During hypoxia, the GRK2 accumulation in mitochondria induced by PH1 protects the mitochondrial mass, so the displacement of GRK2 within cellular compartments is a protective mechanism to preserve mitochondrial integrity, which is essential for energy production and cellular survival under low-oxygen conditions. Conversely, it has previously been demonstrated that in macrophages, LPS inflammation increases mitochondrial GRK2, which promotes biogenesis and recovers mitochondrial function, highlighting how GRK2 localization is a mechanism employed by the cell to preserve mitochondria in response to acute stimuli [22].

Our results further enhance the concept of targeting GRK2 in CVDs, such as during ischemia. Future studies are needed to deepen the GRK2 protective effects, particularly its interaction with mitochondrial pathways and antioxidant defenses during acute and stressful conditions. Additionally, studies in vivo are warranted to validate these findings and establish the therapeutic efficacy of GRK2 modulation in CVD animal models.

## Materials and methods

### Endothelial cells isolation

ECs were isolated from C57BL/6N male mice according to Wang et al. [45]. The Local Ethics Committee at the University of Salerno (n° AUT.N.211/2019-PR) approved the procedures. The thoracic aorta was removed, cleaned of adipose and connective tissue, and placed in cold PBS. Aorta segments (1 mm) were seeded with the endothelium facing downward into a growth factor-reduced matrix and cultured in EC growth media for 4 days. After removing the segments, ECs were cultivated until they reached confluence. The isolation was confirmed using CD31 antibody (anti-mouse PE) (Miltenyi Biotec, Germany, 130-111-540). In total, 400,000 cells were seeded in a p60 dish, harvested with 0.25% Trypsin–EDTA, washed with staining buffer (phosphate buffered saline 1× (PBS), 2% fetal bovine serum (FBS), 0.01% sodium azide), and incubated with CD31 (0.15 μg/μl) at 37 °C for 30 min. The pellet was resuspended in staining buffer, and data analysis was assessed using FACSCalibur software (BD Biosciences, Meylan, France).

### Cell culture and hypoxia induction

ECs were cultured in Dulbecco’s Modified Eagle Medium (DMEM) supplemented with 10% FBS and 1% penicillin–streptomycin at 37 °C in a 5% CO_2_ humidified incubator. Hypoxia was induced by a hypoxic chamber (1% O2, 5% CO_2_, 94% N_2_) for 30 min.

### Animal model

Swine femoral arteries were isolated as “ex vivo material” (Local Ethics Committee at the University of Salerno n° 142/2022-PR) and kept in a tube with cold Krebs–Henseleit’s solution (Sigma-Aldrich, Missouri, United States).

### GRK2 mitochondrial co-localization

The carboxyl-terminal pleckstrin homology domain of GRK2 can promote its accumulation in mitochondria [22]. Furthermore, using a previously cloned strategy [25], a small peptide (10 aa) derived from the GRK2 PH domain, PH 1 (sequence: VPKMKNKPRSGRKKRRQRRRPPQ-TAT conjugated, Neobiotech, Seoul, South Korea) or the scrambled sequence was used to evaluate the GRK2 re-localization in the mitochondria.

### Immunofluorescence

ECs were treated with PH1 (1 μM) or scrambled for 1 h. Cells were treated with Mitotracker Red (400 nM) for 30 min and then fixed with 4% paraformaldehyde. After permeabilization with 0.005% saponin, cells were incubated with primary anti-GRK2 antibody (1:50, Invitrogen, Waltham, Massachusetts, United States, MA5-15840) for 2 h, followed by an Alexa Fluor 488-conjugated secondary antibody (1:200, Vector Lab, United States, DI 2488) for 1 h. Fluorescent images were acquired using a confocal microscope (Leica TCS SP5) at 63× magnification.

### Cytosolic/mitochondrial extracts

Mitochondrial and cytosolic fractions of ECs were isolated according to the manufacturer’s instructions (Thermo Scientific, Waltham, Massachusetts, United States). GRK2 re-localization was analyzed by Western blotting using GRK2 (1:500, Invitrogen MA5-15840) and the ATP synthase β subunit antibodies (1:500 as a mitochondrial marker, Sigma-Aldrich, MABS1304). Band intensities were analyzed using the ChemiDoc MP system (Bio-Rad, California, United States).

### Vascular reactivity studies

Swine femoral artery segments were utilized for vascular reactivity, as previously described [46]. Vessels were mounted in a pressure myograph system filled with Krebs-Henseleit’s solution (pH 7.4 at 37 °C in oxygenated 95% O_2_/5% CO_2_), and isometric tension was recorded. Basal vasoconstrictive response was evaluated with KCl (80 mmol/L), followed by precontraction with phenylephrine (10^−9^ M to 10^−6^ M), and dose-response curves to acetylcholine (Ach, 10^−9^ M to 10^−6^ M) were assessed. GRK2 translocation was induced by incubating the vessels with PH1 (0.1 µM) for 1 h and hypoxia by removing oxygen for 5 min. Vessels were washed with Krebs solution, pre-contracted with phenylephrine, and dose-response curves to Ach were evaluated post-hypoxia.

### ROS analysis in femoral arteries

Swine femoral arteries were snap-frozen with OCT embedding compound. Transverse cryosections (8 μm) were produced using a cryostat (Leica CM1950, Germany), and reactive oxygen species (ROS) were determined using dihydroethidium staining (DHE, Sigma-Aldrich). Sections were washed in PBS and incubated with DHE (5 µM) for 30 min at 37 °C. Images were acquired under a Nikon Eclipse Ti-E fluorescence microscope (Nikon) at 20× magnification. Fluorescence intensity was analyzed with ImageJ software (United States).

### Western blot analysis

Cells were lysed in the RIPA lysis buffer system (Santa Cruz Biotechnology, Dallas, Texas, United States). Samples were separated by polyacrylamide gel 10% Bis–Tris and transferred to PVDF membranes. Primary antibodies for Hypoxia-inducible factor 1-alpha (HIF-1α, Santa Cruz Sc-13515), Superoxide dismutase 2 (SOD-2, Santa Cruz, sc-137254), Xanthine oxidase (Santa Cruz, sc-398548), Oxidative phosphorylation (OXPHOS, Abcam, Cambridge, United Kingdom, M5504300), and Peroxisome proliferator-activated receptor gamma coactivator 1-alpha (PGC-1α, Santa Cruz, sc-518038), Glyceraldehyde 3-phosphate dehydrogenase (GAPDH, Sigma-Aldrich, MAB374) 1:1000 were incubated at 4 °C overnight. Band intensities were analyzed using the ChemiDoc MP system (Bio-Rad).

### Cytotoxicity assay

ECs were seeded 10,000 cells/well in 96-well plates. After 24 h, ECs were treated with PH1 (1 μM) for 1 h and subjected to hypoxia for 30 min. CCK-8 solution (CCK-8, Dojindo Laboratories, Rockville, MD, USA, 18 ng/mL) was added to the cell suspension and incubated for 1 h at 37 °C, followed by absorbance measurement (450 nm) using a TECAN plate reader (Männedorf, Switzerland).

### Cellular growth curves

ECs were seeded at 20,000 cells/well in a 12-well plate. Cells were preincubated for 1 hour with PH1 (1 μM). Hypoxia was induced for 30 min, and the cells were cultured for 96 h. After 24-, 48-, 72-, and 96 h, cells were harvested with 0.25% Trypsin–EDTA and counted with LUNA cell counter (Logos Biosystems, United States).

### Scratch test

The scratch assay was performed as described by Liang et al. [47]. Cells were seeded at 40,000 cells/well in a 6-well plate and preincubated with PH1 (1 µM) for 1 h, followed by hypoxia for 30 min. The scratch was made vertically using a tip. Images were taken under a Nikon Eclipse Ti-E microscope (Nikon, Milan, Italy), basally and 6 h post-hypoxia, magnification 4×. Migration was quantified as a percent (%) scratch area using Image J software.

### Apoptosis assays

Apoptosis was evaluated by FACSCalibur (BD Biosciences) using Annexin V/propidium iodide staining (Thermo Scientific, Waltham, Massachusetts, United States). Briefly, cells were preincubated with PH1 (1 µM) for 1 h at 37 °C and then exposed to hypoxia for 30 min. Cells were harvested with 0.25% Trypsin–EDTA, washed with PBS, and stained according to the manufacturer’s instructions. Data acquisition was performed using FACSCalibur software (BD Biosciences).

### Oxidative stress

Cells were seeded at 20,000 cells/well in a 96-well black plate. After 24 h, cells were pre-incubated with PH1 (1 µM) for 1 h and then exposed to hypoxia for 30 min. Mitochondrial ROS were measured using the fluorescent probe MitoSox (Thermo Scientific, 5 µM) for 30 minutes at 37 °C, followed by fluorescence measurement using a TECAN plate reader at 510 nm (excitation) and 580 nm (emission). Cells were incubated with Dihydrorhodamine 123 (DHR) (Sigma-Aldrich, 10 μM) for 30 min to measure intracellular ROS, followed by fluorescence measurement at 500/536 nm.

### Nonyl acridine orange staining

Mitochondrial content was measured using Nonyl Acridine Orange staining (NAO, Thermo Scientific), which binds cardiolipin, a component of the mitochondrial membrane. In total, 200,000 cells were seeded in a p60 dish. After 24 h, cells were preincubated with PH1 for 1 h (1 μM) at 37 °C and exposed to hypoxia for 30 min. Cells were harvested with 0.25% Trypsin–EDTA and washed with PBS 1×. NAO dye (24 nM) was added to cells for 30 min at 37 °C. The pellet was resuspended in PBS, and mitochondrial mass was assessed by FACSCalibur (BD Biosciences).

### Mitochondrial membrane potential

Mitochondrial membrane potential was measured based on mitochondrial accumulation of tetramethylrhodamine methyl ester (TMRM, Thermo Scientific). Cells were seeded at 20,00,000 cells in a p60 dish. After 24 h, cells were preincubated with PH1(1 uM) for 1 h at 37 °C and exposed to hypoxia for 30 min. Cells were harvested with 0.25% Trypsin–EDTA, washed with PBS, and stained for 30 min at 37 °C with TMRM (20 nM). After centrifugation and resuspension in PBS, cells were immediately analyzed by FACSCalibur (BD Biosciences).

### Statistical analysis

Data were expressed as mean ± standard error of the mean (SEM) of at least 3 independent experiments. Statistical analysis was performed using Prism—GraphPad (Boston, United States). *Two-way ANOVA* followed by Tukey’s post hoc test for multiple comparisons, or an unpaired *Student t-test* was used. A *p*-value < 0.05 was considered statistically significant.

## Supplementary information

Figure S1

original blot

## Data Availability

Original data are available upon request. The full-length, uncropped original Western blots are shown in the ‘Supplementary Material’.
