# Supplementary material for: Mitochondrial accumulation of GRK2 as a protective mechanism against hypoxia-induced endothelial dysfunction
Source: Cell Death Discov. 2025 Jul 14;11:324. doi: 10.1038/s41420-025-02628-0 (PMC12259972; doi:10.1038/s41420-025-02628-0)
Supplement: Supplementary file 1 — Figure S1 [file 41420_2025_2628_MOESM1_ESM.pdf]

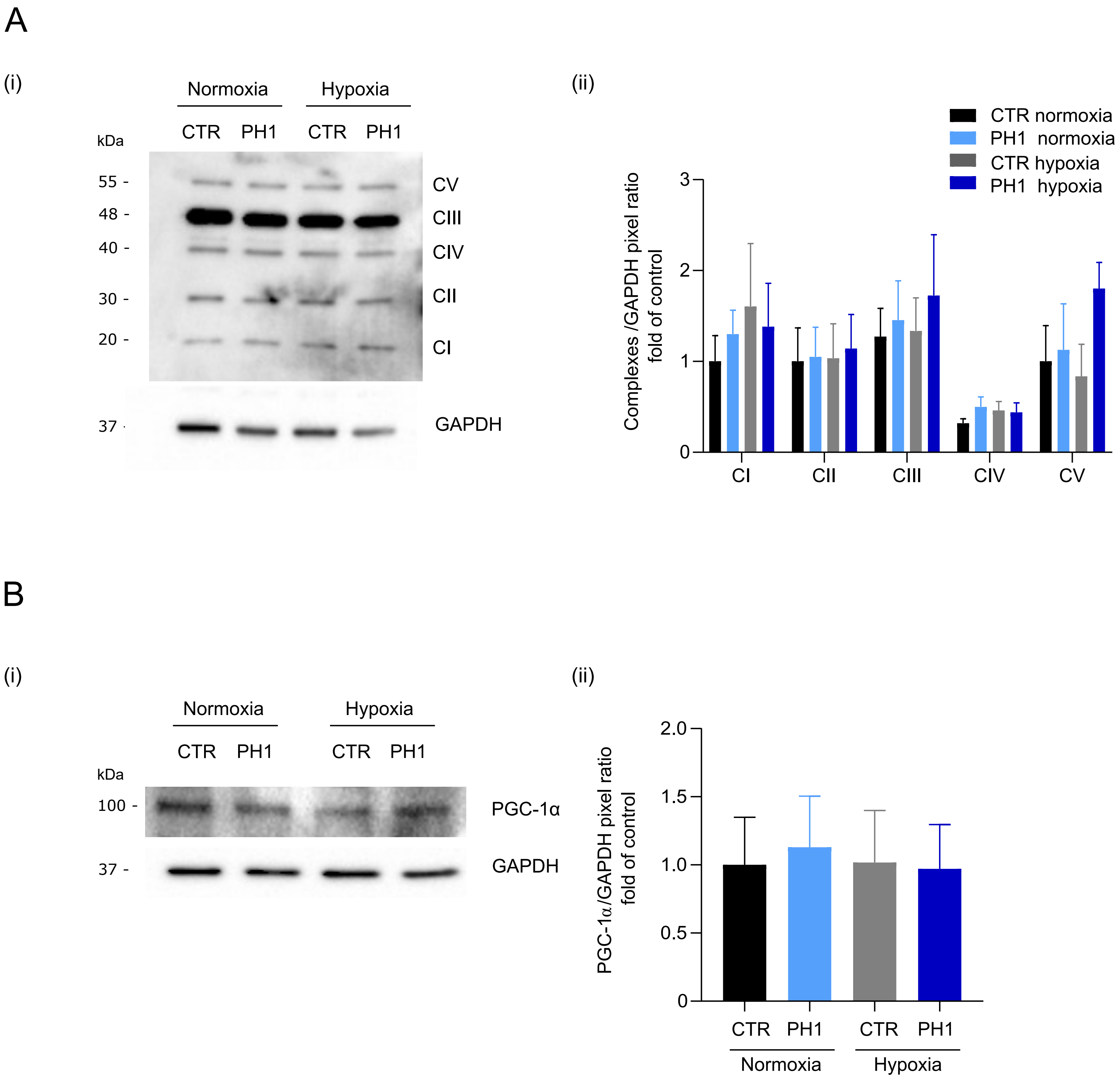
A
(i)
Normoxia
Hypoxia
CTR
PH1
CTR
PH1
kDa
55 -
48 -
40 -
30 -
20 -
37
CV
CIII
CIV
CII
CI
GAPDH
(ii)
CTR normoxia
PH1 normoxia
CTR hypoxia
PH1 hypoxia
Complexes /GAPDH pixel ratio
fold of control
0
1
2
3
CI
CII
CIII
CIV
CV
B
(i)
Normoxia
Hypoxia
CTR
PH1
CTR
PH1
kDa
100 -
37 -
PGC-1α
GAPDH
(ii)
PGC-1α/GAPDH pixel ratio
fold of control
0
0.5
1.0
1.5
2.0
CTR
PH1
CTR
PH1
Normoxia
Hypoxia

Figure S1: In A, western blotting shows (i) the expression of electron transport chain complexes (OXPHOS) and glyceraldehyde 3-phosphate dehydrogenase (GAPDH) as loading control in CTR and PH1-treated cells. Quantification (ii) shows band pixels between OXPHOS and GAPDH. In B, western blotting shows (i) the expression of Peroxisome proliferator-activated receptor gamma coactivator 1-alpha (PGC-1α) and glyceraldehyde 3-phosphate dehydrogenase (GAPDH) as loading control in CTR and PH1-treated cells. Quantification (ii) shows band pixels between PGC-1α and GAPDH. The molecular marker is indicated on the left. Data are the mean ± SEM of 3 independent experiments.
