# Supplementary figures and images for: Mitochondrial accumulation of GRK2 as a protective mechanism against hypoxia-induced endothelial dysfunction
Source: Cell Death Discov. 2025 Jul 14;11:324. doi: 10.1038/s41420-025-02628-0 (PMC12259972; doi:10.1038/s41420-025-02628-0)

Figure 1 A

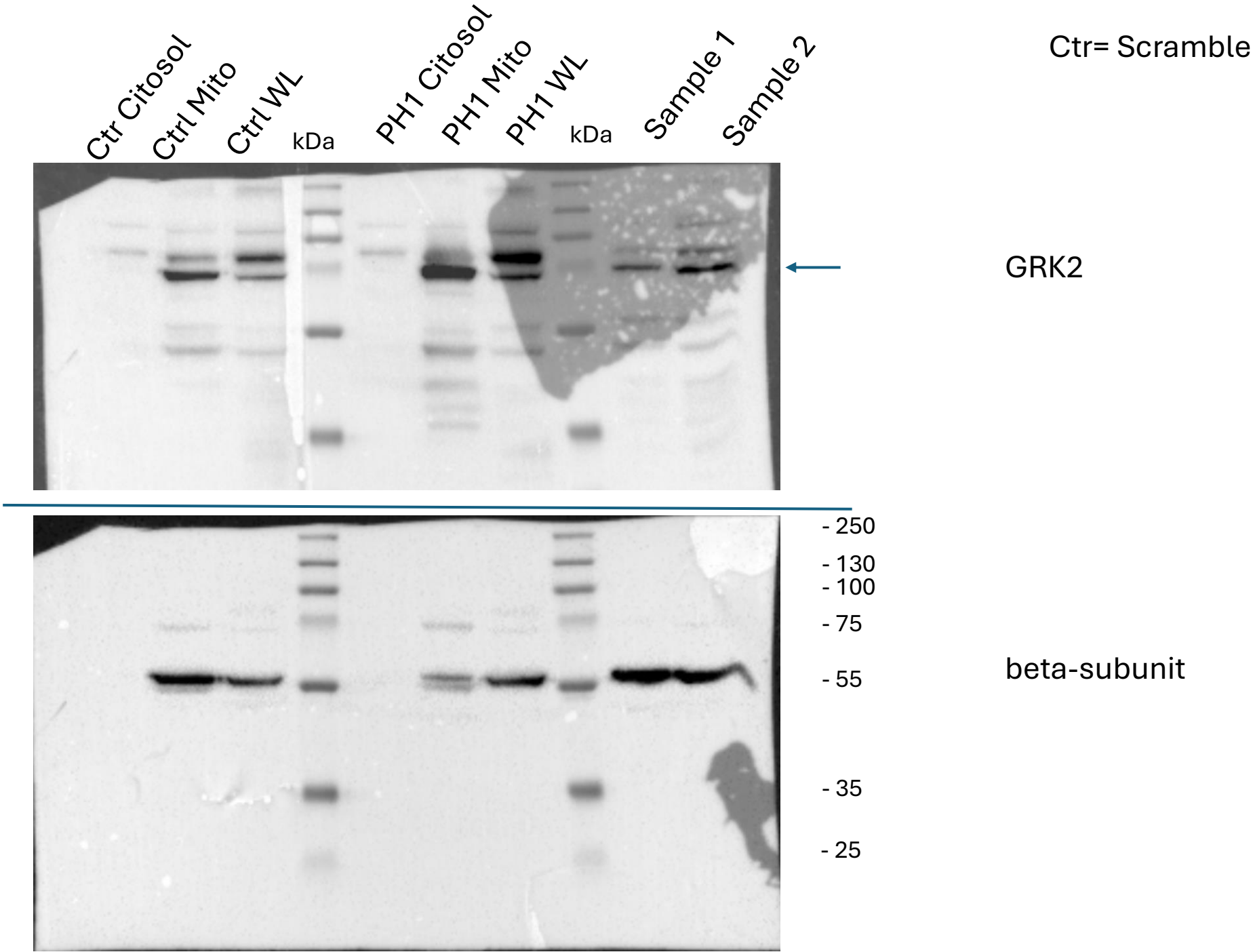

Figure 4 B

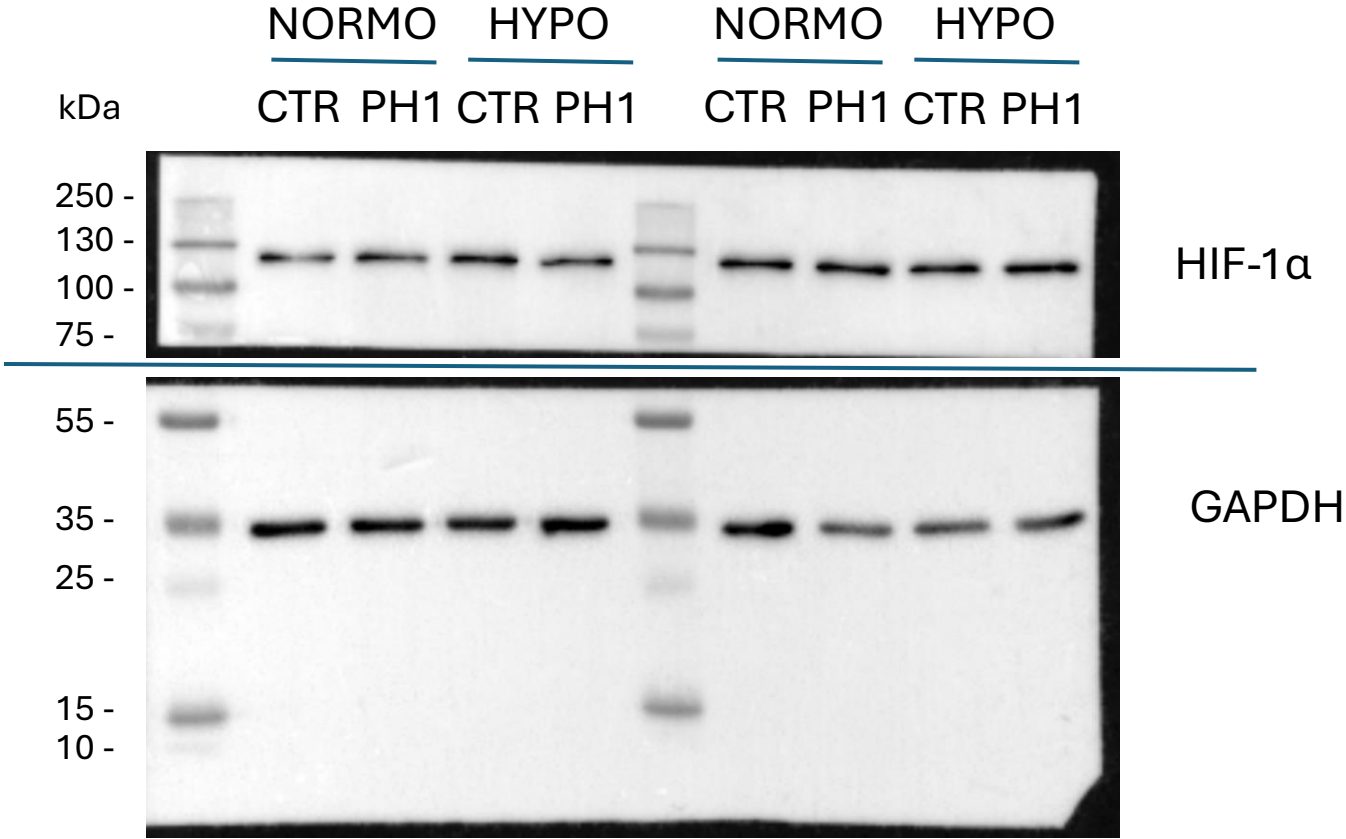

Figure 6 C

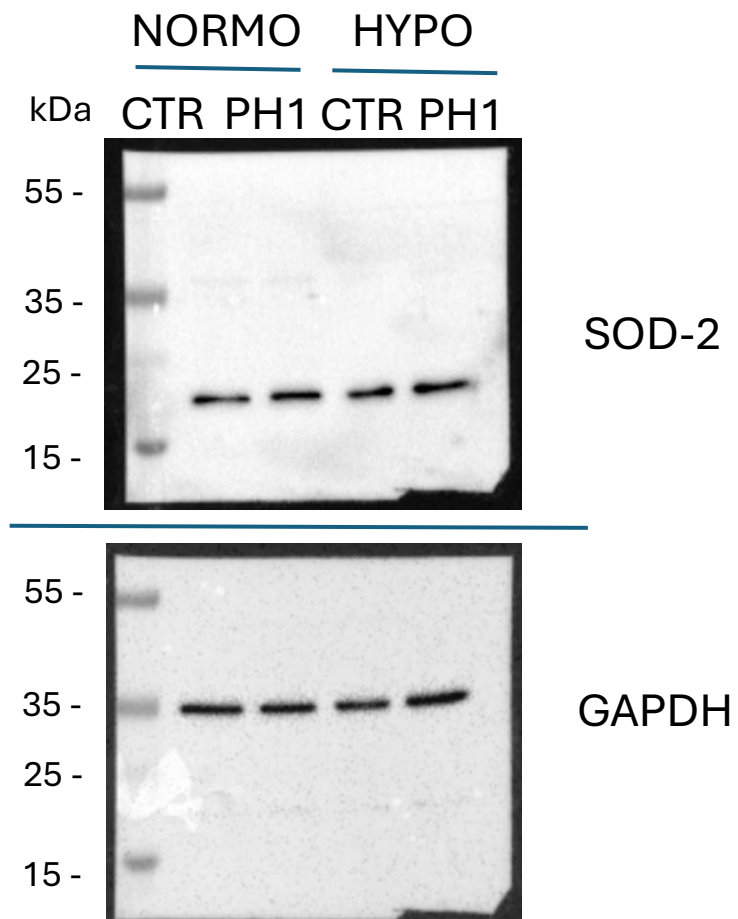

Figure 6 D

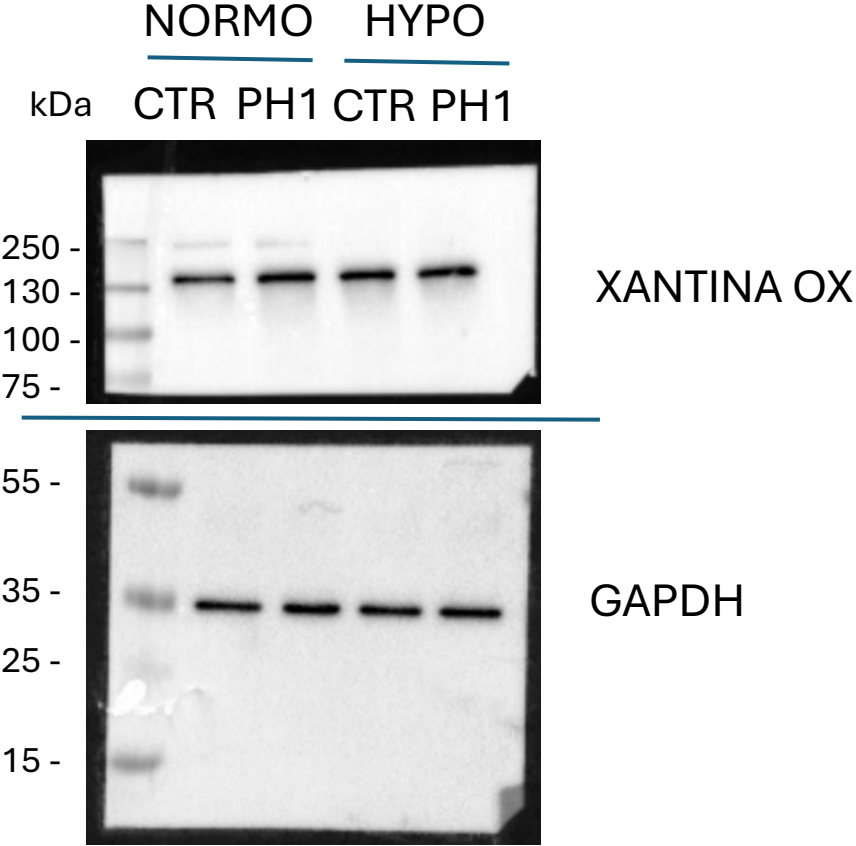

## Figure S1 A

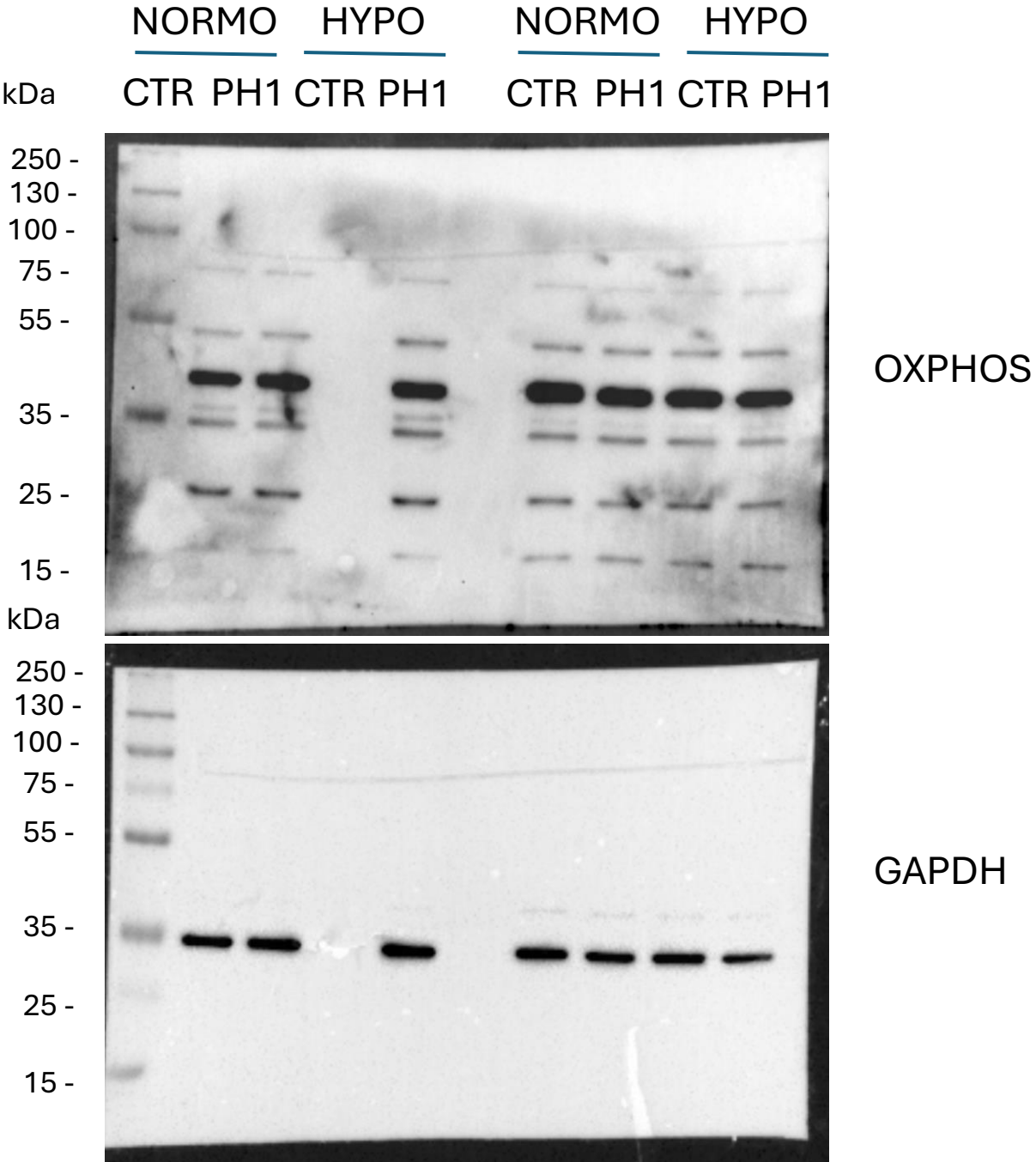

Figure S1 B

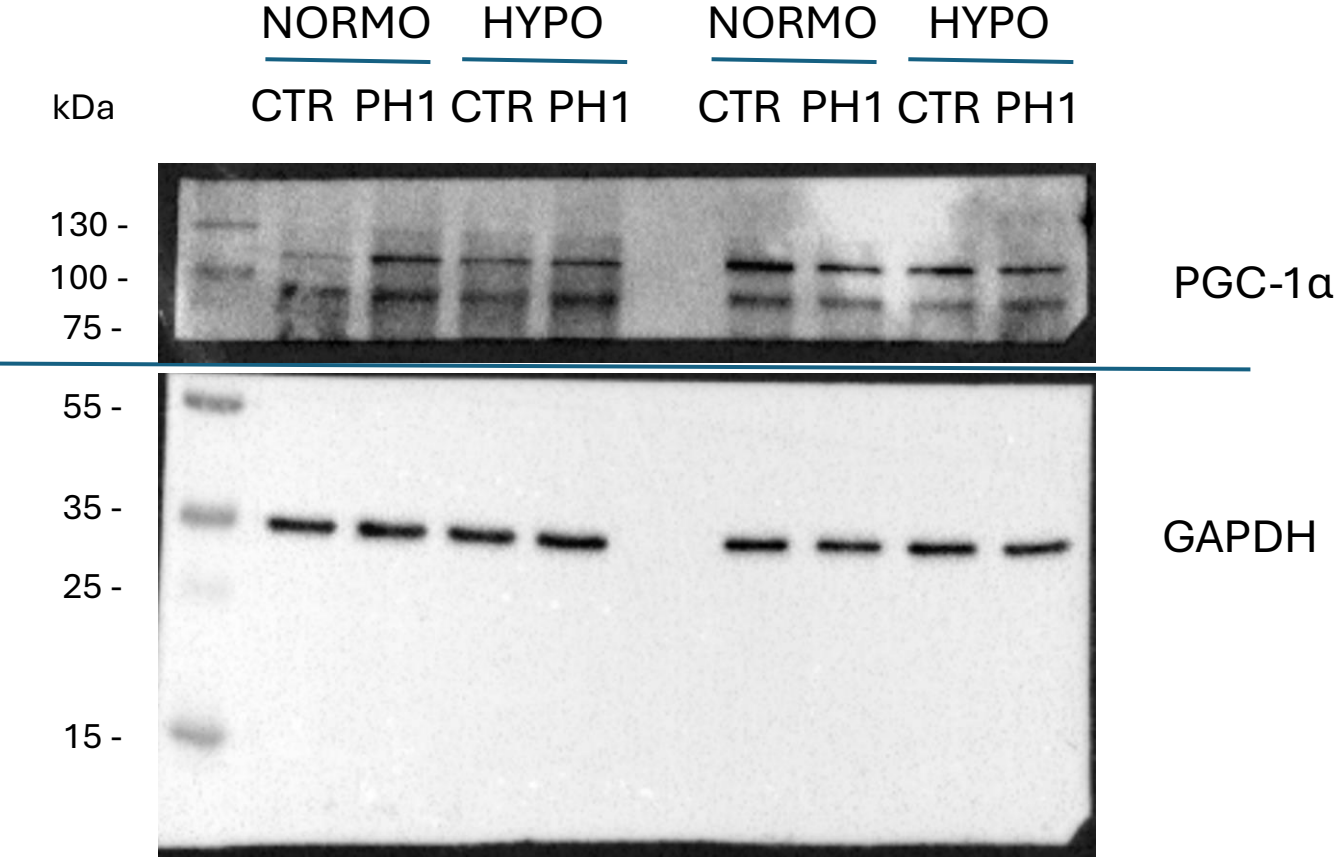

Supplement: Supplementary file 2 — original blot [file 41420_2025_2628_MOESM2_ESM.pdf]
